# Radiographically Occult Carcinomatous Spread of Breast Cancer to the Liver: A Challenging Case

**DOI:** 10.1155/2019/4935615

**Published:** 2019-12-06

**Authors:** Trish Millard, Akriti Gupta, Christiana Brenin, Paul Marshall, Patrick Dillon

**Affiliations:** ^1^Division of Hematology/Oncology, University of Virginia, 1215 Lee St, Charlottesville, VA 22908, USA; ^2^Department of Pathology, University of Virginia, 1215 Lee St, Charlottesville, VA 22908, USA

## Abstract

Metastatic carcinomatosis to the liver is a pattern of malignant infiltration that tends to provoke hepatic fibrosis. It is a rare complication of multiple solid tumor types and often seen in the absence of discrete tumor mass in the liver. We report a case of a 69-year-old woman with metastatic ductal carcinoma of the breast who presented with rising serum tumor markers and mildly cirrhotic contour of the liver on a CT scan. An early diagnosis of occult spread to the liver was made by means of a percutaneous liver biopsy showing desmoplastic response to invasive breast cancer cells found diffusely in the liver. This case highlights a rare pattern of metastatic spread of breast cancer as well as predisposing and prognostic features.

## 1. Case Presentation

We present a 69-year-old Caucasian woman who had a history of metastatic, estrogen receptor-positive, and human epidermal growth factor receptor 2/neu (HER2) nonamplified, invasive ductal breast cancer, and she went on to develop occult liver involvement. The patient originally underwent left modified radical mastectomy in 2010 for a 3.1 cm mass, and she had an axillary nodal dissection which found one of seventeen lymph nodes involved. She was treated with adjuvant Taxotere and Cytoxan chemotherapy for 6 cycles and then completed adjuvant external beam radiation therapy to the chest wall and axilla in 25 fractions. The patient took two years of adjuvant aromatase inhibitor therapy and stopped due to arthralgia.

The patient presented to her oncologist with new pain in the pelvis 5 years after the initial diagnosis (March 2015). A bone scan and CT scan revealed widespread metastatic disease limited to the bones. A biopsy of the left iliac crest confirmed metastatic ductal adenocarcinoma of breast origin which remained 100% positive for the estrogen receptor and 100% positive for the progesterone receptor and negative for HER2. She attempted first-line therapy with palbociclib and letrozole; however, this was stopped for neutropenic fever and osteomyelitis. She was then treated sequentially with letrozole and Faslodex for 35 months, until February 2019 with serial stability on CT scans every 3 months. She received bone strengthening therapy with denosumab throughout her course.

**Figure 1:**
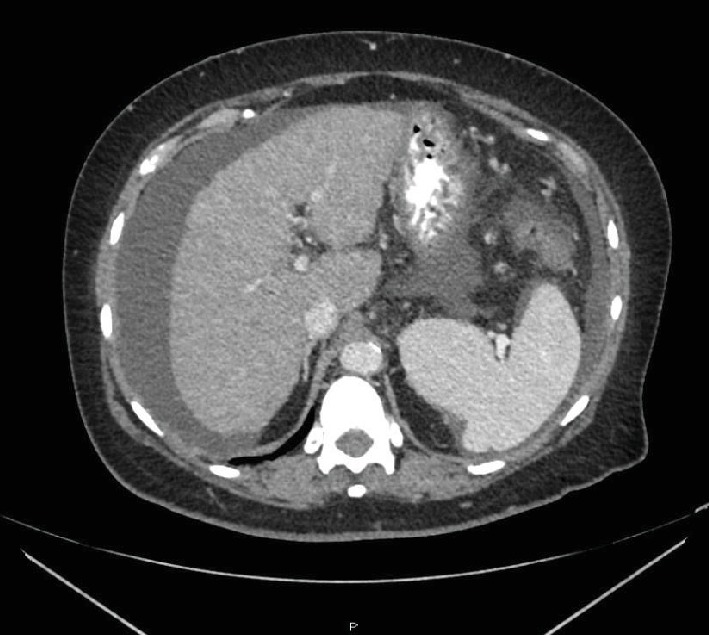
CT demonstrating ascites and mildly nodular liver surface contour (oral and IV contrast present).

Then, at the nine-year mark from her original breast cancer (2/2019), a routine follow-up CT scan (Figure 1) revealed a mildly nodular liver surface contour suggestive of cirrhotic changes, but no focal hepatic lesion. The physical examination revealed no icterus, hepatomegaly, or splenomegaly. There were no stigmata of chronic liver disease and no asterixis. The chest portion of the CT revealed a few small peribronchovascular nodules in the inferior left lower lobe and stable vertebral body bone lesions. The laboratory data at the same time revealed that the serum bilirubin rose to 2.5 mg/dL from a baseline of 1.0 mg/dL two months prior. The alkaline phosphatase rose to 343 U/L from 180; the aspartate aminotransferase (AST) and alanine aminotransferase (ALT) remained within normal limits at 40 and 21, respectively. The albumin was 3.0 g/dL, the PT was 14.6 s (normal is 9-13), the PTT was 39.1 (normal is 27.8-37.6), and the conjugated bilirubin was 1.0 (0-0.5 mg/dL). The serum level of cancer antigen (CA 15-3) rose from 285 to 381 U/mL. Alpha fetoprotein was 7 and CA-125 was 4. Other tumor markers were not checked at the time of the evaluation.

**Figure 2:**
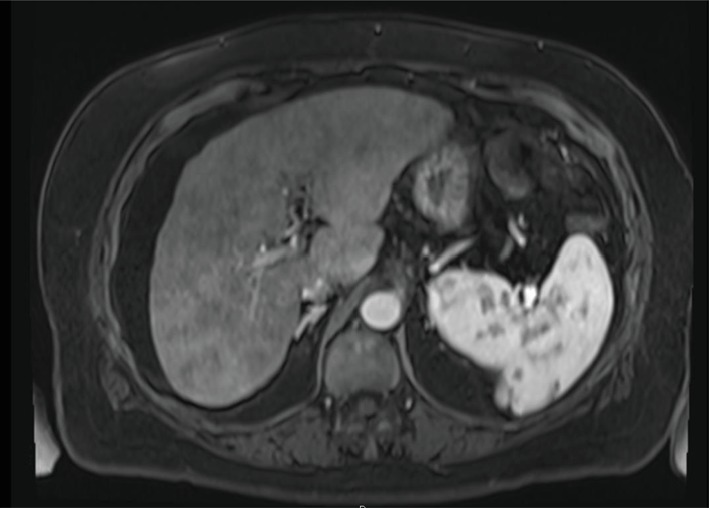
MRI demonstrating course texture (VIBE axial image post gadolinium).

Upon finding evidence of a suddenly cirrhotic appearance of the liver in the absence of known liver disease, the patient underwent evaluation for primary and secondary causes of cirrhosis. She had a negative workup for hepatitis A, B, C and HIV. She had normal iron studies, except for an elevated ferritin of 1,102 ng/mL. She was a nondrinker and nonsmoker who did not use herbal medications or drugs and had not received hepatotoxic agents. She had no international travel, chemical exposures, or farm work. She did not report any insect or animal exposures and she had no sick contacts. She had no family history of liver disease, hemochromatosis, Wilson's disease, or alpha-1 antitrypsin. She was seen by a hepatologist who tested immunoglobulins, erythrocyte sedimentation rate (ESR), and antinuclear antibody to rule out autoimmune hepatitis. The autoimmune panel was only notable for a mildly elevated ESR of 50 (normal 0-30), but that finding was blamed on known metastatic cancer to bones. The hepatologist did not deem her likely to have CMV, EBV, or other viral etiology given lack of extrahepatic findings on CT and lack of symptoms/fevers/weight loss/lymphadenopathy and lack of immunosuppression. An ultrasound of the liver was performed and failed to detect a focal liver lesion, gallstones, biliary obstruction, or abnormal blood flow. She next had gadolinium-enhanced magnetic resonance imaging (MRI) of the liver (Figure 2), which demonstrated a nodular liver surface contour and a fibrotic appearance of the hepatic parenchyma. No masses were detected within the liver. The abdominal vasculature was normal in caliber and all the arteries, veins, and portal vessels were patent. New ascites was seen.

**Figure 3:**
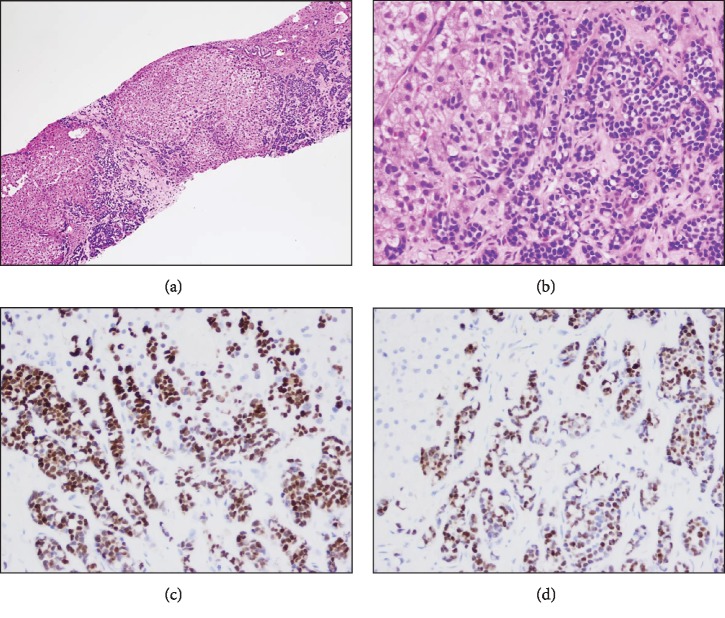
Diffusely infiltrating nests of intermediate-grade ductal adenocarcinoma with disrupted hepatic parenchyma (H&E) ((a) 100x and (b) 400x). Tumor cell nests staining for estrogen (c) and progesterone (d) receptors.

Given the lack of a mass in the liver and lack of intrinsic liver disease found by any serology or lab testing, there was concern for radiographically occult metastasis to the liver. Thus, the patient next underwent a random percutaneous liver biopsy under ultrasound guidance. The random liver biopsy revealed liver parenchyma disrupted by a desmoplastic tissue response that was diffusely infiltrated by nests of intermediate grade ductal adenocarcinoma with multiple intracytoplasmic lumens which recapitulated breast tissue appearance. Immunohistochemical stains for estrogen receptor and progesterone receptor demonstrated positivity in 100% and 90% of tumor cells, respectively (Figure 3). An additional immunohistochemical (IHC) stain with mammaglobin (not shown) was 1+ positive, further supporting a breast origin for the malignant cells. HER2 was not amplified by FISH. The morphology and IHC stains were completely concordant with the original tumor morphology and receptors. Ascites fluid at the same time was cytologically negative for malignant cells. Serial scans did not detect other primary cancer aside from the breast cancer.

The patient was treated with paracentesis for symptom control. She was offered palliative chemotherapy, but ultimately declined additional therapies in order to pursue a comfort-based approach. Given her preference for palliative care, the patient did not undergo esophagogastroduodenoscopy (EGD) screening for varices.

## 2. Discussion

The liver is a common location of metastatic disease in solid tumor oncology. Hepatic metastases usually demonstrate discrete mass lesions and are sometimes painful when they involve the capsule of the liver [1]. On the other hand, descriptions of occult metastatic carcinomatosis of the liver such as characterized in this case report have not been widely reported (see Table 1 for literature review). A few published cases have described a diffuse infiltration of the liver by ductal or lobular breast cancer but often not found until the time of an autopsy [2–6]. The first report of occult, diffuse liver invasion of any histology was described by Watson in 1955 involving several cases of disseminated bronchogenic carcinoma leading to fulminant liver failure [7]. Since that time, carcinomatosis or sinusoidal infiltration of the liver (also termed hepar lobatum carcinomatosum) has been reported due to primary tumors of the colon, pancreas, lung, stomach, kidney, adrenal, ovary, breast, and lymphomas [8]. Occult involvement of the liver due breast cancer has been described only in case reports and has been too rare to formally study [2, 3, 5, 6, 9–18]. Unfortunately, no authors have been able to elucidate common risk factors nor common shared biologic features (Table 1). Loss of E-cadherin appears common, but our case like others had histologic evidence of the breast cancer cells attempting to recapitulate round ductal structures which is not typically seen in breast cancer which is lacking E-cadherin [9, 16]. It is believed that breast cancers downregulate (or lose) E-cadherin as cells from the primary tumor gain access to the angiolymphatic system in the process of epithelial to mesenchymal transition (EMT). Cancerous cells may then reexpress E-cadherin at the metastatic niche by mesenchymal to epithelial transition (MET) [19]. We hypothesize that this is the process that we observed in the liver of our patient. We also point out that other adhesion molecules (VCAM-1, P-cadherin, and ICAMs and selectins such as E-selectin and integrins such as LFA-a and VLA-4) and chemokine receptors (CXCR4, CCR6, and CCR7) have been speculated to be involved in the homing of breast cancer cells to the liver and warrant additional research.

This case report highlights the challenging clinical diagnosis of occult liver involvement by breast cancer when radiographic imaging fails to detect any mass forming lesions in the liver. It should be pointed out that rapidly progressive liver failure in estrogen receptor-positive breast cancer is atypical and warrants a thorough diagnostic workup. Commonly, the cross-sectional imaging done for metastatic breast cancer demonstrates either discrete lesions within the liver or occasionally the sequelae of regressed hepatic lesions with capsular retraction and decrease in the liver size. Indeed, liver involvement by breast cancer has often only been able to be identified postmortem owing to the occasional lack of definitive radiographic evidence and perhaps also due to the indolent rate of progression of some estrogen-driven breast cancers [4]. Detection of liver metastases may also be delayed in the modern era of CDK4/6 therapy whereby the interval of cross-sectional imaging may be extended beyond the historic three-month timeframe. We recommend performing MRI of the liver when breast cancer patients first demonstrate inexplicable rises in liver function tests or any other abnormality of the liver on standard CT imaging. The MRI may be followed by liver biopsy whenever liver enzymes remain elevated in the setting of inexplicable liver contour changes in a metastatic breast cancer patient.

Our literature review suggests that extensive liver involvement is sometimes seen in breast cancer patients after prolonged intervals without overt disease progression. Subtle signs of liver involvement may include weight changes, subclinical bleeding from varices, slow accumulation of ascites, jaundice, and even slowly progressive hepatic encephalopathy [13, 20]. In retrospect, there is suspicion that our case of occult liver infiltration may have developed subtly over 6-12 months with the only indicator being subtle increases in bilirubin and alkaline phosphatase values to less than one time the upper limit of normal. It is worth pointing out that there are also existing reports of de novo diagnosis of breast cancer made upon finding occult hepatic involvement [11, 14].

Regardless of whether metastatic liver carcinomatosis is an early or late complication of breast cancer, the prognosis is currently deemed to be quite poor and is generally less than 6 months overall and less than 3 months when evidence of hepatic failure is present [6, 9, 12]. Treatment options are limited due to the fact that most cytotoxic chemotherapy is difficult to administer in the setting of hepatic impairment. Likewise, the CDK4/6 inhibitors and the mTOR inhibitors are not recommended in Child-Pugh class C hepatic impairment. Liver transplant is currently not indicated for patients with diffuse metastatic disease.

## 3. Conclusion

Liver metastases of breast cancer usually present as discrete metastatic nodules and diagnosis is often straightforward on cross-sectional imaging, but can occasionally present as radiographically occult disease. Unfortunately, the diagnosis of carcinomatous spread within the liver is difficult to make and is often delayed. In cases of cirrhotic-appearing liver such as the one described here, a high level of suspicion is required and a liver biopsy is essential to making the diagnosis. We have not been able to discern specific predisposing features for this pattern of metastatic spread. Both ductal and lobular histologies have been observed to follow this pattern. Neither receptor status nor E-cadherin status has correlated with the risk of diffuse liver invasion. Unfortunately, this phenotypic pattern of metastatic breast cancer has been poorly studied to date. The treatment options are limited and the prognosis is currently viewed as poor.

## Figures and Tables

**Table 1:** Summary of literature reports of diffuse and/or occult liver involvement by metastatic breast cancer.

Authors	Age	Receptors	Concurrent diagnoses/presentation/notes	E-cad loss	Imaging	Time from 1° Dx	Histology	Ref
Jungst et al., 2013	70	NA	Diabetes, variceal bleeding	NA	Cirrhosis on MRI, no focal lesion	7 yr	Ductal+lobular	[13]
Allison et al., 2004	42	ER-, PR-	Tobacco abuse, thrombocytopenia	Yes	Not performed	0 yr	Poorly differentiated	[9]
Allison et al., 2004	54	NA	Ascites, jaundice, hematochezia	Yes	Multiple masses on CT	9 yr	Poorly differentiated	[9]
Allison et al., 2004	36	NA	Nausea/vomiting/abd pain and jaundice	Yes	Small masses on CT	9 mo	Poorly differentiated	[9]
Hanamornroongruang and Sangchay, 2013	49	ER+, PR+	Weight loss, jaundice, abd pain	Yes	CT without mass	10 yr	Ductal, stage I	[2]
Martelli et al., 2000	53	ER+, PR+	Dyspnea, fever	NA	Hepatomegaly on US	4 yr	Ductal	[3]
Mogrovejo et al., 2014	67	ER+, PR-, HER2-	Fatigue, weakness, abdominal distention/jaundice, lower extremity edema	No	CT ascites	21 yr	Ductal+lobular	[4]
Borja et al., 1975	46	NA	Jaundice, ascites, spider angiomata	NA	CT cirrhosis	3 mo	Poorly differentiated	[5]
Graber et al., 2010	57	ER+, PR+, HER2+	Hyperthyroid, ascites, thrombocytopenia, hyperbilirubinemia	Yes	CT/US w/o mass, heterogeneous parenchyma	0 yr	Lobular	[6]
Fournier et al., 2010	52	ER+, PR-, HER2-	Bone mets from breast cancer, abdominal fullness	NA	CT with ascites, splenomegaly, but no liver abnormality	5 yr	Ductal, stage II	[10]
Goswami et al., 2011	38	ER-, PR+, HER2+	Malaise, lethargy, sore throat, dyspnea	NA	CT hepatomegaly, periportal nodes	0 yr	Ductal, grade 2	[11]
Morrison and Pennington, 1984	57	NA	Rapid fulminant hepatic failure	NA	NA	0 yr	Carcinoma NOS	[14]
Nakajima et al., 2005	68	NA	Drowsiness, disorientation, lymphangitic cancer spread to lungs	NA	CT normal liver, laparoscopic slight cirrhosis	8 mo	Ductal, stage I	[15]
Nascimento et al., 2001	62	NA	Ascites, edema	NA	US w/o mass, reversal of portal flow, MRI w cirrhosis	13 yr	NA	[16]
Nascimento et al., 2001	46	NA	Nausea, vomiting, abdominal girth increase, disseminated intravascular coagulopathy	NA	CT neg for lesions, MRI w cirrhosis	5 yr	Mucinous, poorly differentiated	[16]
Sass et al., 2007	55	ER+, PR-, HER2-	Jaundice, dark urine, acholic stool, and abdominal distension	NA	CT nodular contour c/w cirrhosis, no focal lesion	4 yr	Ductal, stage I	[17]
Shivashankar and Sweetser, 2012	66	ER+, PR+	Abdominal distention, hepatomegaly	NA	CT with cirrhosis, but no mass	8 yr	NA	[18]
Current case	69	ER+, PR+, HER2-	Bone mets from breast cancer, no presenting symptom, eventual leptomeningeal metastases	Yes	Cirrhosis on CT, US and MRI	9 yr	Ductal	

NA: not available; E-cad: E-cadherin; ER: estrogen receptor; PR: progesterone receptor; Ref: reference number; NOS: not otherwise specified; CT: computerized tomography; MRI: magnetic resonance imaging.

## References

[1] Koenigsberg M., Freeman L. M. (1975). Multinuclide evaluation of hepatic mass lesions. *CRC Critical Reviews in Clinical Radiology and Nuclear Medicine*.

[2] Hanamornroongruang S., Sangchay N. (2013). Acute liver failure associated with diffuse liver infiltration by metastatic breast carcinoma: a case report. *Oncology Letters*.

[3] Martelli O., Coppola L., de Quarto A. L., Palma M., Sarmiento R., Foggi C. M. (2000). Fulminant hepatic failure caused by diffuse intrasinusoidal metastatic liver disease: a case report. *Tumori Journal*.

[4] Mogrovejo E., Manickam P., Amin M., Cappell M. S. (2014). Characterization of the syndrome of acute liver failure caused by metastases from breast carcinoma. *Digestive Diseases and Sciences*.

[5] Borja E. R., Hori J. M., Pugh R. P. (1975). Metastatic carcinomatosis of the liver mimicking cirrhosis: case report and review of the literature. *Cancer*.

[6] Graber I., Dumortier J., Poncet G., Queneau P. E., Mathevet P., Scoazec J. Y. (2010). Hepar lobatum carcinomatosum revealing an occult metastatic lobular carcinoma of the breast. *Annals of Diagnostic Pathology*.

[7] Watson A. J. (1955). Diffuse intra-sinusoidal metastatic carcinoma of the liver. *The Journal of Pathology and Bacteriology*.

[8] Jha P., Poder L., Wang Z. J., Westphalen A. C., Yeh B. M., Coakley F. V. (2010). Radiologic mimics of cirrhosis. *American Journal of Roentgenology*.

[9] Allison K. H., Fligner C. L., Parks W. T. (2004). Radiographically occult, diffuse intrasinusoidal hepatic metastases from primary breast carcinomas: a clinicopathologic study of 3 autopsy cases. *Archives of Pathology & Laboratory Medicine*.

[10] Fournier C., Tisman G., Kleinman R., Park Y., Macdonald W. D. (2010). Clinical evidence for overcoming capecitabine resistance in a woman with breast cancer terminating in radiologically occult micronodular pseudo-cirrhosis with portal hypertension: a case report. *Journal of Medical Case Reports*.

[11] Goswami R., Babich M., Farah K. F. (2011). Occult breast malignancy masquerading as acute hepatic failure. *Gastroenterology & hepatology*.

[12] Gulia S., Khurana S., Shet T., Gupta S. (2016). Radiographically occult intrasinusoidal liver metastases leading to hepatic failure in a case of breast cancer. *BMJ Case Reports*.

[13] Jungst C., Kramer J., Schneider G., Lammert F., Zimmer V. (2013). Subacute liver failure by pseudocirrhotic metastatic breast cancer infiltration. *Annals of Hepatology*.

[14] Morrison W. L., Pennington C. R. (1984). Liver metastases from an occult breast carcinoma presenting as acute fulminant hepatic failure. *The British Journal of Clinical Practice*.

[15] Nakajima T., Sekoguchi S., Nishikawa T. (2005). Multifocal intraportal invasion of breast carcinoma diagnosed by laparoscopy-assisted liver biopsy. *World Journal of Gastroenterology*.

[16] Nascimento A. B., Mitchell D. G., Rubin R., Weaver E. (2001). Diffuse desmoplastic breast carcinoma metastases to the liver simulating cirrhosis at MR imaging: report of two cases. *Radiology*.

[17] Sass D. A., Clark K., Grzybicki D., Rabinovitz M., Shaw-Stiffel T. A. (2007). Diffuse desmoplastic metastatic breast cancer simulating cirrhosis with severe portal hypertension: a case of “pseudocirrhosis”. *Digestive Diseases and Sciences*.

[18] Shivashankar R., Sweetser S. (2012). A woman with ascites, nodular liver contour, and splenomegaly. *Gastroenterology*.

[19] Palen K., Weber J., Dwinell M. B., Johnson B. D., Ramchandran R., Gershan J. A. (2016). E-cadherin re-expression shows *in vivo* evidence for mesenchymal to epithelial transition in clonal metastatic breast tumor cells. *Oncotarget*.

[20] Knouse P., Hancock C., Iwaz S., Kaiser P. (2019). Metastatic carcinomatosis cirrhosis: a rare pattern of metastasis. *Cureus*.

